# Anthropometric Analysis of Brazilian and Imported Total Knee Arthroplasty Implants in the Brazilian Population

**DOI:** 10.1055/s-0045-1812025

**Published:** 2025-11-21

**Authors:** Márcio de Castro Ferreira, Carlos Eduardo da Siveira Franciozi, Luiz Felipe Morlin Ambra, Enzo Salviato Mameri, Marcelo Seiji Kubota, Marcus Vinícius Malheiros Luzo

**Affiliations:** 1 Knee Surgery Group, Department of Orthopedics and Traumatology, Escola Paulista de Medicina, Universidade Federal de São Paulo, São Paulo, SP, Brazil.; 2 HCor, São Paulo, SP, Brazil.

**Keywords:** arthroplasty, replacement, joint disease, knee joint, articulação do joelho, artroplastia de substituição, doenças articulares

## Abstract

**Objective:**

To evaluate the anatomical conformity of 25 total knee arthroplasty (TKA) implants to the knee morphology of the Brazilian female and male population.

**Methods:**

We analyzed 500 magnetic resonance imaging (MRI) scans of knees from 250 women and 250 men. We collected data on anteroposterior (AP) and mediolateral (ML) measurements of femurs, tibias, and implants to assess their morphological correspondence.

**Results:**

The mean AP versus ML divergence between joint morphologies and the implants was as follows: 4.48 mm for female femurs; 4.89 mm for male femurs; 3.63 mm for female tibias; and 6.11 mm for male tibias. The implants with the best AP versus ML adaptation were: Medacta Sphere for female femurs; Stryker Triathlon for male femurs; Smith & Nephew Legion for female tibias; and Zimmer Persona for male tibias. When comparing the best femoral and tibial implant ratios for female subjects, the United Orthopedic U2 presented the best statistical score, followed by the Aesculap Columbus and Smith &Nephew Legion implants. For male patients, the implants with the highest scores were the Zimmer Persona, the Microport Advance, and the Smith & Nephew Legion. The worst ratios were found in the Peter Brehm BPK-S for female individuals and the Orthovasive Indus for male subjects.

**Conclusion:**

The implants studied presented satisfactory results for bone coverage of the knees of Brazilian subjects of both genders undergoing TKA. However, we also found differences higher than 10 mm in most implants. This finding highlights that surgeons must carefully plan the TKA during implant selection. Imported implants proved to be more customizable than Brazilian ones in this population.

## Resumo

**Objetivo:**

Avaliar a conformidade anatômica de 25 implantes de artroplastia total de joelho (ATJ) à morfologia de joelhos na população brasileira feminina e masculina.

**Métodos:**

Foram analisados 500 exames de ressonância de joelhos de 250 mulheres e 250 homens. As medidas anteroposterior (AP) e mediolateral (ML) dos fêmures, tíbias, e implantes foram tomadas a fim de obter correspondência morfológica.

**Resultados:**

As divergências médias das medidas AP e ML entre as morfologias articulares e os implantes analisados foram as seguintes: fêmures femininos 4,48 mm; fêmures masculinos 4,89 mm; tíbia feminina 3,63 mm; e tíbia masculina 6,11 mm. Os implantes com melhor adaptação AP versus ML foram: Medacta Sphere para o fêmur feminino; Stryker Triathlon para fêmur o masculino; Smith & Nephew Legion para tíbia feminina; e Zimmer Persona para a tíbia masculina. Na comparação da melhor relação de implantes femorais e tibiais para o sexo feminino, observou-se que o United Orthopedic U2 apresentou a melhor pontuação estatística, seguido dos implantes Aesculap Columbus e Smith & Nephew Legion. Para os homens, o melhor escore foi encontrado no Zimmer Persona, Microport Advance e Smith & Nephew Legion. A pior relação feminina foi com o Peter Brehm BPK-S, e a masculina, com o Orthovasive Indus.

**Conclusão:**

Os implantes estudados foram satisfatórios para a cobertura óssea articular dos joelhos de brasileiros de ambos os sexos submetidos à ATJ. No entanto, também foram encontrados resultados com diferenças maiores do que 10 mm na maioria dos implantes. Isso ressalta a necessidade de atenção dos cirurgiões para realizar um bom planejamento para a escolha dos implantes. Demonstrou-se que os implantes importados eram mais customizáveis do que os nacionais na população brasileira.

## Introduction

Total knee arthroplasty (TKA) is the treatment of choice for patients with advanced osteoarthritis. 1 2 3 4 5 6 The sizing of implants for TKA is based on studies of the bone morphology of population groups, mainly the anatomical articular ratios between the anteroposterior (AP) and mediolateral (ML) distances in the knees. 7 8 9 10

Studies in several global populations, including Caucasians, Americans, Asians, and Europeans, reported variable results for the morphological aspects of the knees, demonstrating that the available implants may differ from the anatomical relationships in diverse ethnic populations. 7 11 12 13 14

The present study aimed to evaluate the relationship between the anatomical conformity of TKA implants and knee morphology in the Brazilian population, to identify which devices best suit the knees of our patients.

## Materials and Methods

The two ethics committees of the institutions involved approved this study under numbers CAAE 71751923.0.0000.5505 and 79484424.0.0000.0060. We randomly selected 500 anonymous magnetic resonance imaging (MRI) scans of the knees of 250 women and 250 men.

The inclusion criterion was skeletal maturity of male or female patients. The exclusion criteria were scans showing morphological knee bone deformities (osteophytes), implantable medical devices, and evidence of previous joint fractures.

We identified the eligible patients through the MRI database of the Hospital Hcor in its Clinical Collaboration Platform and software (Carestream Health, Inc.). We analyzed only the right knee from patients with bilateral imaging.

Using the software, we measured the ML and AP distances from the femur and tibia, from axial, T2-weighted MRI scans obtained using a method validated for the Brazilian population: 1

**Fig. 1:**
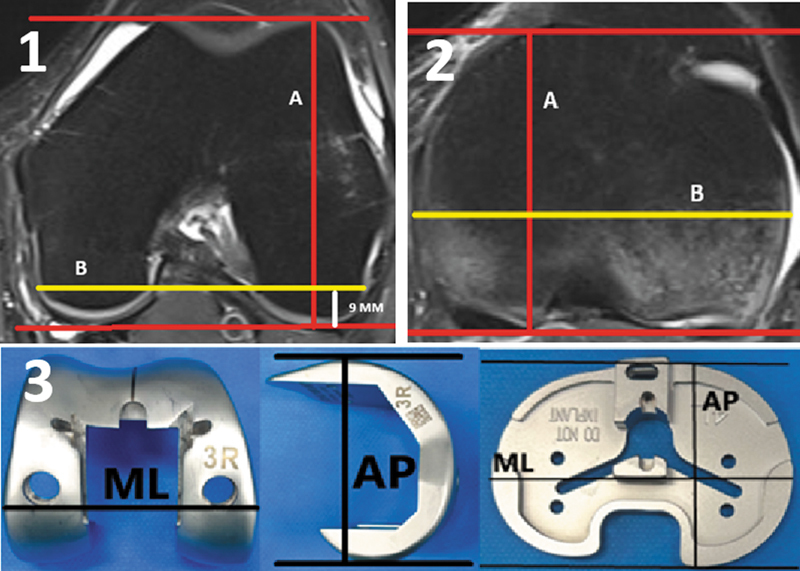
Anteroposterior (AP) measurement ( **A** ) per the most anterior and posterior articular prominence of the lateral femoral condyle and bicortical mediolateral (ML) distance ( **B** ) 9 mm from the femoral posterior articular surface at the level of the epicondylar axial cut. AP measurement ( **C** ) according to the most anterior and posterior articular prominence of the tibial plateau and ML distance ( **D** ) at the largest bicortical axis of the tibial articular surface at the level of the axial section of the posterior cruciate ligament (PCL) attachment. AP and ML measurements ( **E** ) from femoral and tibial arthroplasty implants.

- Femoral AP dimension: the greatest distance in the AP axis at the lateral condyle between the most prominent posterior and anterior articular ends ( Fig. 1A ).
- Femoral ML dimension: distance from the bicortical axis to 9 mm from the posterior joint line of the knee ( Fig. 1B ).
- Tibial AP dimension: the largest axis perpendicular to the posterior cortex of the medial tibial plateau at the level of the tibial PCL ( Fig. 1C ).
- Tibial ML dimension: the greatest distance between the medial and lateral tibial cortices parallel to the posterior cortex at the level of the tibial posterior cruciate ligament (PCL) ( Fig. 1D ).

A single knee surgeon performed all measurements twice, on different days. We included only measurements showing a variation lower than 10% in the intraobserver analyses.

We searched for international TKA implant manufacturers on the Orthopaedic Data Evaluation Panel's website (ODEP; https://www.odep.org.uk/methodology/methodology-for-tkr/ ). All international and Brazilian manufacturers were contacted by email. We also searched for information on their commercial websites to obtain data on the ML and AP measurements of their TKA implants. The measurements corresponded to the axis of greatest AP and ML prominence of the implants in the two planes indicated by the manufacturers ( Fig. 1E ).

We identified 20 manufactures and 25 implants: Columbus (Aesculap AG), AKS (Baumer), Sigma and Attune (DePuy Synthes), ACS (Implantcast GmbH), NG and PAR (Impol Instrumental e Implantes Ltda.), Gemini and Symphoknee (Waldemar Link GmbH & Co. ), Freedom (Maxx Orthopedics), Sphere (Medacta Corp.), Freedom (Meril Life), Advance (Microport Orthopedics Inc.), Orthovasive Indus (Biorad Medisys Pvt Ltd.), Ortosíntese (Ortosintese), BPK-S (Peter Brehm GmbH), Sartori (Sartori), Legion (Smith + Nephew), Scorpio and Triathlon (Stryker), U2 (United Orthopedic Corp.), MetaBio and Rotaflex (Vincula), as well as Persona and Nex Gen (Zimmer Biomet).

### Statistical Analysis

We analyzed and compared epidemiological data regarding gender, age, and morphological dimensions expressed as mean and standard deviation (SD) values.

To determine the most appropriate femoral implant from each manufacturer for each patient, we prioritized the closest AP distance, which could be equal, undersized, or oversized. The ML reference measurement identified the most appropriate tibial components. The mean analyses included both undercoverage and overcoverage values for the AP and ML measurements.

We used the Euclidean distance to correlate the femur and tibia dimensions to the prosthesis dimensions, comparing their proximity in a two-dimensional space. The Euclidean distance formula was the following:



We analyzed the mean and maximum variations, SDs, and statistical difference ( *p* -value) of the relationships between the anatomical and implant measurements. The analysis of variance (ANOVA) test determined the existence of significant differences between the types of prostheses. The Shapiro-Wilk and Levene tests verified the assumptions of normality and homogeneity of variances. The significance level was set at *p* = 0.05.

We aggregated AP and ML measurements of the femoral and tibial implants to rank the devices according to their match with the knees of our patients. As such, implants with the smallest differences from bone morphologies received higher scores. To create a ranking of the prosthetic implants, we normalized the values to the range of 0 to 1, then established a score (normalized tibial value and normalized femur value). Sample calculation considered a 90% confidence level.

## Results

We assessed 500 patients (250 men and 250 women). Their mean age was 51 years, with an average of 53 years for women and 49 years for men ( Table 1 ).

**Table 1:** Epidemiological data and morphological statistical analysis of the femoral mediolateral (ML) and anteroposterior (AP) measurements

	Total (mean)	Male gender (mean)	Female gender (mean)
**Patients (n)**	500	250	250
**Age (years)**	50.91 ± 14.76	48.82 ± 15.43	53.01 ± 13.73
**ML: femur (mm)**	72.11 ± 5.93	76.22 ± 4.83	68.00 ± 3.65
**AP: femur (mm)**	67.34 ± 5.03	69.92 ± 5.14	64.74 ± 3.30
**ML: tibia (mm)**	73.63 ± 6.48	76.89 ± 6.86	70.36 ± 3.94
**AP: tibia (mm)**	53.02 ± 4.94	56.23 ± 4.45	49.81 ± 2.92

For men, the mean femoral AP measurement was of 69.92 ± 5.14mm, and the mean femoral ML width was of 76.22 ± 4.83 mm. Women presented a mean AP measurement of 64.74 ± 3.30 mm, and a mean ML width of 68.00 ± 3.65 mm ( Table 1 ).

For women, the average divergence between the implants and the femoral AP/ML ratios was 4.23 mm. The average variations for the AP measurements were 0.98 mm, with 1.11 mm for anatomies larger than the implants, and 0.91 mm for implants larger than the anatomy. The mean ML measurements were 3.94 mm, with the anatomy larger than the implants by an average of 1.75 mm, and 4.15 mm for implants larger than the anatomy. Most implants had ML measurements larger than the femurs. The best morphological measurement ratios were found in Medacta Sphere, with a mean ML/AP divergence of 2.36 ± 1.66 mm ( Table 2 ).

**Table 2:** Comparison of femoral morphological data and knee implants regarding mediolateral (ML) and anteroposterior (AP) measurements in female subjects

	Euclidian distance (AP and ML)					ML measurements								AP measurements							
Manufacturer; prosthesis	Mean (mm)	Min (mm)	Max (mm)	SD	*p* -value	Mean (mm)	SD	Max (mm)	Prosthetic < Patient's AP (n)	Mean prosthetic < Patient's AP (mm)	Prosthetic > Patient's AP (n)	Mean prosthetic > Patient's AP (mm)	Mean prosthetic = Patient's AP (mm)	Mean (mm)	SD	Max (mm)	Prosthetic ML < Patient's ML (n)	Mean prosthetic < Patient's ML (mm)	Prosthetic > Patient's ML (n)	Mean prosthetic ML > Patient's ML (mm)	Prosthetic ML = Patient's ML (n)
Medacta; Sphere	2.36	0.20	8.45	1.66		0.46	0.55	1.00	116	0.54	114	0.46	20	2.25	2.82	8.4	133	2.38	112	2.19	5
Unites Orthopedic; U2	2.36	0.00	8.34	1.69	1.000	0.54	0.61	1.00	128	0.60	108	0.54	14	2.22	2.76	8.3	96	1.99	149	2.43	5
Link; Symphoknee	2.39	0.14	7.30	1.41	1.000	0.79	0.90	1.50	123	0.82	117	0.83	10	2.11	2.41	7.3	105	1.29	142	2.77	3
Aesculap; Columbus	2.39	0.00	7.28	1.39	1.000	1.11	1.30	2.50	138	1.20	102	1.10	10	1.91	2.24	7	174	2.05	72	1.69	4
Implantcast; ACS Knee System	2.53	0.10	7.45	1.44	1.000	0.95	1.14	3.50	113	1.11	126	0.89	11	2.18	2.10	7.4	206	2.34	40	1.58	4
Link; Gemini	2.72	0.28	10.30	1.88	0.989	0.83	1.00	3.00	132	0.93	109	0.77	9	2.42	2.67	10.3	71	1.32	176	2.91	3
Maxx Orthopedics; Freedom	2.76	0.00	7.45	1.65	0.969	0.85	0.99	2.00	140	0.91	101	0.84	9	2.46	3.00	7.4	104	2.25	141	2.71	5
Meril; Freedom	2.79	0.00	7.45	1.67	0.930	0.85	0.99	2.00	138	0.91	102	0.84	10	2.49	3.03	7.4	104	2.25	141	2.76	5
DePuy; Attune	3.09	0.32	10.20	1.87	0.050	0.78	0.91	1.60	109	0.83	133	0.79	8	2.86	2.83	10.1	55	1.83	192	3.20	3
Microport; Advance	3.36	0.22	11.44	1.96	0.000	1.00	1.14	2.00	120	1.05	121	1.02	9	3.08	2.83	11.3	43	1.95	205	3.35	2
DePuy; Sigma	3.45	0.32	11.44	1.77	0.000	1.09	1.38	6.00	128	1.31	110	0.97	12	3.06	3.16	11.3	71	2.29	176	3.42	3
Zimmer; Persona	3.47	0.36	9.30	1.97	0.000	0.83	0.94	1.90	121	0.87	120	0.85	9	3.26	2.80	9.3	39	1.84	210	3.54	0
Stryker; Triathlon	3.49	0.36	9.30	2.01	0.000	0.79	0.90	1.50	121	0.84	119	0.81	10	3.30	2.83	9.3	39	1.84	210	3.58	1
Orthovasive; Indus	3.61	0.36	9.30	2.29	0.000	1.44	1.88	8.00	122	1.72	126	1.19	2	3.05	3.26	9.3	63	2.05	185	3.42	2
Smith & Nephew; Legion	3.97	0.22	11.30	2.18	0.000	0.67	0.81	2.00	129	0.78	105	0.63	16	3.83	3.01	11.3	34	2.04	215	4.13	1
Stryker; Scorpio	4.06	0.20	11.33	2.21	0.000	0.93	1.21	5.00	137	1.15	98	0.77	15	3.77	3.05	11.3	35	1.92	214	4.10	1
Peter Brehm; BPK-S	4.22	0.73	12.26	2.18	0.000	1.43	1.60	3.00	149	1.46	95	1.46	6	3.77	3.28	12.1	39	2.37	210	4.05	1
Vincula; Rotaflex	4.31	0.22	11.60	2.55	0.000	0.44	0.52	1.50	133	0.53	95	0.42	22	4.24	3.31	11.6	34	1.92	215	4.63	1
Impol; PAR	4.88	1.08	12.61	2.09	0.000	1.68	2.14	7.00	114	2.01	128	1.49	8	4.25	2.76	12.3	19	1.69	228	4.52	3
Impol; NG	5.99	0.94	13.32	2.79	0.000	1.14	1.50	7.00	131	1.43	112	0.87	7	5.68	3.48	13.3	17	2.04	232	5.97	1
Zimmer; Nex Gen	6.66	0.36	13.30	2.77	0.000	1.44	1.92	9.00	146	1.80	100	0.96	4	6.21	3.27	13.3	10	1.49	239	6.44	1
Sartori; Sartori	7.14	0.36	13.30	3.09	0.000	1.07	1.28	5.00	129	1.18	117	0.97	4	6.95	3.27	13.3	3	0.70	247	7.03	0
Baumer; Baumer	7.56	1.25	14.32	3.07	0.000	1.05	1.31	5.00	118	1.13	125	1.04	7	7.40	3.24	14.3	3	1.23	247	7.48	0
Vincula; MetaBio	7.73	0.14	13.50	3.05	0.000	1.10	1.30	4.50	133	1.24	111	0.99	6	7.58	3.16	13.4	2	0.30	248	7.64	0
Ortosintese; Ortosintese	8.35	1.08	17.95	2.94	0.000	1.28	1.51	4.00	145	1.40	93	1.28	12	8.15	3.09	17.8	2	0.30	248	8.22	0

Abbreviations: Max, maximum value; Min, minimum value; SD, standard deviation.

For men, the mean divergence in femoral AP/ML measurements was 4.71 mm. The mean variations for AP measurements were 1.94 mm, with 2.39 mm for anatomies larger than the implants and 0.92 mm for implants larger than the anatomy. For ML measurements, the mean divergence was 3.81 mm, with the anatomy larger than the implants by an average of 4.02 mm and implants larger than the anatomy by 2.87 mm. Most implants had ML measurements smaller than the anatomies. The best morphological measurement ratios were found in Triathlon, with a mean divergence in the ML/AP ratio of 3.5 ± 2.69 mm ( Table 3 ).

**Table 3:** Comparison of femoral morphological data and knee implants regarding mediolateral (ML) and anteroposterior (AP) measurements in male subjects

	Euclidian distance (AP and ML)					ML measurements								AP measurements							
Manufacturer; prosthesis	Mean (mm)	Min (mm)	Max (mm)	SD	*p* -value	Mean (mm)	SD	Max (mm)	Prosthetic < Patient's AP (n)	Mean prosthetic < Patient's AP (mm)	Prosthetic > Patient's AP (n)	Mean prosthetic > Patient's AP (mm)	Mean prosthetic AP = Patient's AP (mm)	Mean (mm)	SD	Max (mm)	Prosthetic < Patient's ML (n)	Mean prosthetic < Patient's ML (mm)	Prosthetic > Patient's ML (n)	Mean prosthetic > Patient's ML (mm)	Prosthetic ML = Patient's ML (n)
Stryker; Triathlon	3.52	0.14	13.61	2.69		1.00	1.67	10.00	143	1.28	99	0.68	8	3.16	3.94	13.6	145	3.62	102	2.59	3
Zimmer; Persona	3.55	0.14	13.61	2.68	1.000	1.15	1.77	10.00	141	1.39	102	0.90	7	3.12	3.89	13.6	150	3.60	97	2.48	3
Microport; Advance	3.63	0.41	15.22	2.50	1.000	1.29	1.77	9.00	102	1.63	143	1.10	5	3.19	3.79	15.1	158	3.61	92	2.47	0
Link; Symphoknee	3.64	0.36	12.61	2.61	1.000	0.92	1.37	8.00	138	1.09	106	0.76	6	3.33	3.59	12.6	177	3.95	70	1.88	3
DePuy; Attune	3.65	0.14	15.16	2.81	1.000	1.02	1.41	7.70	136	1.16	109	0.89	5	3.32	4.12	15.1	141	4.26	108	2.13	1
Smith & Nephew; Legion	3.68	0.10	14.61	2.47	1.000	1.02	1.68	10.00	156	1.24	84	0.73	10	3.34	4.05	14.6	122	3.74	128	2.96	0
Aesculap; Columbus	3.73	0.14	15.22	2.54	1.000	1.11	1.33	4.50	137	1.20	111	1.01	2	3.39	3.72	15.1	196	3.55	54	2.79	0
Meril; Freedom	4.03	0.36	15.61	3.27	0.993	1.04	1.70	10.00	152	1.27	89	0.77	9	3.61	4.42	15.6	160	4.42	87	2.24	3
United Orthopedic; U2	4.08	0.36	15.61	3.34	0.974	1.05	1.83	11.00	152	1.42	87	0.54	11	3.72	3.90	15.6	192	4.35	56	1.67	2
Implantcast; ACS Knee System	4.10	0.32	16.66	2.86	0.961	1.39	1.61	6.00	168	1.54	76	1.16	6	3.60	3.47	16.6	224	3.77	25	2.27	1
Peter Brehm; BPK-S	4.15	0.22	15.46	2.37	0.906	1.43	1.88	8.50	114	1.64	133	1.29	3	3.63	4.36	15.3	102	3.75	146	3.60	2
Medacta; Sphere	4.19	0.22	17.12	3.43	0.839	0.67	1.23	8.00	119	0.89	116	0.53	15	4.02	3.97	17.1	207	4.53	40	1.71	3
Maxx Orthopedics; Freedom	4.21	0.36	15.61	3.42	0.800	1.27	1.92	11.00	164	1.54	79	0.83	7	3.76	3.99	15.6	189	4.50	57	1.56	4
Vincula; Rotaflex	4.24	0.42	13.61	2.85	0.731	1.05	1.79	11.00	172	1.36	62	0.44	16	3.78	4.51	13.6	83	3.62	165	3.91	2
Link; Gemini	4.64	0.20	17.56	3.08	0.027	2.01	2.70	13.00	148	2.65	96	1.14	6	3.81	3.85	12.6	176	4.65	71	1.87	3
Stryker; Scorpio	4.85	0.10	18.48	3.29	0.001	2.49	3.25	15.00	174	3.20	73	0.90	3	3.59	4.26	14.1	147	4.17	102	2.80	1
Sartori; Sartori	5.24	0.10	17.39	2.86	0.000	2.42	3.22	15.00	175	3.14	68	0.79	7	3.87	4.44	10.1	98	3.31	151	4.26	1
Ortosintese; Ortosintese	5.49	0.14	15.56	2.92	0.000	2.47	3.10	14.00	153	3.25	95	1.26	2	4.21	4.29	11.6	64	2.58	185	4.80	1
Vincula; MetaBio	5.54	0.10	16.02	2.91	0.000	2.36	3.11	14.50	170	3.03	75	1.01	5	4.22	4.38	11.6	69	2.80	180	4.79	1
Impol; PAR	5.64	0.78	20.69	3.71	0.000	3.68	4.09	17.00	192	4.28	56	1.76	2	3.67	4.21	11.8	155	4.28	94	2.71	1
Baumer; Baumer	5.70	0.28	16.47	2.98	0.000	2.42	3.31	15.00	157	3.37	90	0.83	3	4.27	4.41	11.4	74	2.52	175	5.03	1
Impol; NG	5.92	0.22	20.69	3.64	0.000	3.43	3.88	17.00	186	4.34	58	0.86	6	4.08	4.82	11.8	137	4.52	113	3.54	0
DePuy; Sigma	6.21	0.36	21.80	4.51	0.000	2.95	3.48	16.00	199	3.48	44	1.00	7	5.06	4.54	15.6	209	5.67	39	2.09	2
Zimmer; Nex Gen	6.80	0.10	22.37	3.95	0.000	4.73	4.30	19.00	217	5.36	27	0.71	6	3.91	4.65	11.8	131	4.57	119	3.19	0
Orthovasive; Indus	7.30	0.20	23.95	4.84	0.000	4.13	4.16	18.00	199	4.94	46	1.04	5	5.54	4.65	15.8	212	6.14	36	2.30	2

Abbreviations: Max, maximum value; Min, minimum value; SD, standard deviation.

Tibial morphological findings in females demonstrated that the components presented mean ML/AP divergences of 3.63 mm. The Legion implant presented the smallest mean variation, with an ML divergence of 0.88 mm. Virtually half of the women had implants, on average, oversized by 1.33 mm and undersized by 1.26 mm for the tibial ML measurements. The mean AP ratio demonstrated a divergence from the anatomical measurements of 3.14 mm. The mean AP over- and undersize values were 0.85 mm and 3.23 mm, respectively. The Advance implant stood out, with 202 women presenting a mean AP oversize of 3.46 mm ( Table 4 ).

**Table 4:** Comparison of tibial morphological data and knee implants regarding ML and AP measurements in female subjects

	Euclidian distance (AP and ML)					ML measurements								AP measurements							
Manufacturer; prosthesis	Mean (mm)	Min (mm)	Max (mm)	SD	*p* -value	Mean (mm)	SD	Max (mm)	Prosthetic < Patient's ML (n)	Mean prosthetic < Patient's ML (mm)	Prosthetic > Patient's ML (n)	Mean prosthetic > Patient's ML (mm)	Prosthetic = Patient's ML (n)	Mean (mm)	SD	Max (mm)	Prosthetic < Patient's AP (n)	Mean prosthetic < Patient's AP (mm)	Prosthetic > Patient's AP (n)	Mean prosthetic > Patient's AP (mm)	Mean prosthetic = Patient's AP (mm)
Smith & Nephew; Legion	2.19	0.22	17.76	1.55		0.88	1.37	15.8	129	0.82	117	0.97	4	1.85	2.29	13.20	130	2.03	119	1.67	1
Zimmer; Persona	2.54	0.20	16.74	1.61	0.984	1.09	1.46	13.5	130	1.12	115	1.11	5	2.14	2.62	14.00	124	2.28	123	2.04	3
United Orthopedic; U2	2.66	0.10	20.47	2.02	0.740	0.88	1.52	18.8	131	0.88	111	0.96	8	2.34	2.38	14.20	192	2.67	53	1.33	5
Aesculap; Columbus	2.74	0.58	18.82	1.51	0.395	1.37	1.86	17.8	113	1.42	132	1.38	5	2.12	1.37	12.20	246	2.15	3	0.27	1
Maxx Orthopedics; Freedom	2.76	0.14	17.92	1.83	0.330	1.29	1.74	14.8	127	1.23	120	1.38	3	2.19	2.40	13.20	172	2.66	74	1.22	4
Vincula; Rotaflex	2.83	0.14	15.52	1.66	0.134	1.56	1.94	13.8	101	1.63	147	1.54	2	2.11	2.32	13.20	173	2.42	76	1.42	1
Meril; Freedom	2.92	0.20	17.92	1.80	0.029	1.29	1.74	14.8	127	1.23	120	1.38	3	2.39	2.36	13.20	191	2.72	57	1.36	2
Zimmer; Nex Gen	3.31	0.20	15.27	2.20	0.000	1.03	1.46	11.8	111	1.11	132	1.02	7	2.98	2.57	15.20	211	3.34	37	1.11	2
Microport; Advance	3.37	0.20	14.07	1.89	0.000	1.07	1.31	9.8	112	1.03	134	1.13	4	3.03	2.57	10.10	47	1.25	202	3.46	1
Stryker; Triathlon	3.63	0.32	19.60	2.31	0.000	0.93	1.45	16.8	110	0.88	133	1.02	7	3.36	2.41	15.20	235	3.52	15	0.96	0
Orthovasive; Indus	3.63	0.10	17.99	2.41	0.000	1.20	1.84	15.8	121	1.35	123	1.11	6	3.21	2.46	14.20	222	3.55	24	0.62	4
Baumer; Baumer	3.66	0.28	17.10	1.86	0.000	1.92	2.32	13.8	144	1.88	103	2.03	3	2.79	1.90	15.20	250	2.79	0	0.00	0
Impol; NG	3.68	0.20	23.17	2.31	0.000	2.00	2.72	22.8	133	1.99	113	2.07	4	2.73	2.03	15.20	244	2.78	6	0.42	0
Sartori; Sartori	3.68	0.20	19.99	2.04	0.000	1.62	2.12	17.8	146	1.65	100	1.63	4	3.05	1.96	15.20	250	3.05	0	0.00	0
Vincula; MetaBio	3.70	0.20	19.99	2.06	0.000	1.62	2.11	17.8	147	1.67	99	1.61	4	3.07	1.98	15.20	250	3.07	0	0.00	0
Stryker; Scorpio	3.71	0.30	19.60	2.20	0.000	1.10	1.65	16.8	141	1.05	105	1.20	4	3.35	2.29	16.20	236	3.50	12	0.93	2
Ortosintese; Ortosintese	3.80	0.28	15.53	1.84	0.000	1.98	2.33	11.8	146	2.01	102	1.98	2	2.92	1.93	15.20	250	2.92	0	0.00	0
DePuy; Attune	3.81	0.14	18.50	2.24	0.000	0.85	1.30	14.8	121	0.79	123	0.94	6	3.60	2.26	16.50	241	3.72	9	0.47	0
Medacta; Sphere	3.81	0.58	18.75	2.15	0.000	1.38	1.79	15.8	105	1.39	140	1.41	5	3.34	2.39	15.20	230	3.55	18	1.03	2
DePuy; Sigma	3.85	0.10	18.66	2.29	0.000	1.05	1.54	15	112	1.04	132	1.09	6	3.54	2.44	15.40	236	3.69	12	1.21	2
Link; Gemini	3.95	0.22	19.56	2.39	0.000	1.35	1.87	17.8	98	1.28	146	1.45	6	3.44	2.58	15.20	226	3.74	22	0.64	2
Link; Symphoknee	4.35	0.20	19.44	2.37	0.000	0.92	1.36	14.8	115	0.90	128	0.99	7	4.15	2.36	16.20	243	4.26	6	0.42	1
Impol; PAR	4.41	0.22	22.39	2.54	0.000	1.38	2.04	21.8	120	1.26	128	1.52	2	3.99	2.46	16.20	240	4.14	9	0.37	1
Implantcast; ACS Knee System	4.83	0.95	19.90	2.38	0.000	1.04	1.55	15.8	123	1.04	119	1.11	8	4.59	2.35	17.70	246	4.66	3	0.27	1
Peter Brehm; BPK-S	6.35	2.13	20.59	2.48	0.000	1.06	1.51	15.8	113	0.95	130	1.21	7	6.19	2.41	17.90	250	6.19	0	0.00	0

Abbreviations: Max, maximum value; Min, minimum value; SD, standard deviation.

For the male population, the tibial ML and AP ratios showed that the implant with the best morphological measurement ratio was the Persona device, with a mean ML/AP ratio divergence of 3.27 ± 3.00 mm. The mean ML over- and undersize values were 1.77 mm and 1.99 mm respectively. The mean AP ratio had a divergence from the anatomical measurements of 5.54 mm. The overall mean AP over- and undersizing were 0.69 and 5.78 mm respectively ( Table 5 ).

**Table 5:** Comparison of tibial morphological data and knee implants regarding mediolateral (ML) and anteroposterior (AP) measurements in male subjects

	Euclidian distance (AP and ML)					ML measurements								AP measurements							
Manufacturer; prosthesis	Mean (mm)	Min (mm)	Max (mm)	SD	*p* -value	Mean (mm)	SD	Max (mm)	Prosthetic < Patient's ML (n)	Mean prosthetic < Patient's ML (mm)	Prosthetic > Patient's ML (n)	Mean prosthetic > Patient's ML (mm)	Prosthetic = Patient's ML (n)	Mean (mm)	SD	Max (mm)	Prosthetic < Patient's AP (n)	Mean prosthetic < Patient's AP (mm)	Prosthetic > Patient's AP (n)	Mean prosthetic > Patient's AP (mm)	Mean prosthetic = Patient's AP (mm)
Zimmer; Persona	3.27	0.14	17.18	3.00		1.14	1.57	8.2	115	1.01	130	1.29	5	2.90	3.97	15.60	134	3.83	116	1.82	0
Microport; Advance	3.38	0.22	16.18	2.53	1.000	1.03	1.26	4.5	125	1.04	119	1.08	6	3.05	3.90	15.80	67	3.79	181	2.81	2
Smith & Nephew; Legion	3.50	0.10	16.76	3.17	1.000	1.12	1.79	10.5	124	0.93	119	1.39	7	3.19	3.62	13.80	194	3.63	54	1.72	2
Aesculap; Columbus	4.34	0.36	16.55	3.17	0.233	1.43	2.21	12.5	100	1.27	143	1.62	7	3.90	2.94	12.90	250	3.90	0	0.00	0
Vincula; Rotaflex	4.36	0.32	14.84	3.18	0.202	1.40	1.86	8.5	113	1.33	132	1.51	5	3.89	3.51	12.80	220	4.28	27	1.08	3
Stryker; Triathlon	4.39	0.51	18.97	3.53	0.157	1.19	1.96	11.5	124	1.02	120	1.42	6	4.07	3.91	15.80	204	4.67	46	1.42	0
United Orthopedic; U2	4.72	0.42	18.68	3.73	0.006	2.06	3.04	13.5	138	2.35	110	1.75	2	3.97	3.78	13.80	204	4.59	43	1.32	3
Medacata; Sphere	5.28	0.50	18.44	3.58	0.000	1.57	2.11	10.5	97	1.34	148	1.77	5	4.80	3.70	15.80	236	5.05	11	0.86	3
Meril; Freedom	5.49	0.32	17.94	4.19	0.000	2.58	3.21	9.9	164	3.16	82	1.53	4	4.52	4.06	15.80	219	5.03	28	1.01	3
Maxx Orthopedics; Freedom	5.49	0.32	17.94	4.19	0.000	2.58	3.21	9.9	164	3.16	82	1.53	4	4.52	4.06	15.80	219	5.03	28	1.01	3
DePuy; Sigma	5.74	0.89	18.92	3.60	0.000	1.53	2.00	9.7	102	1.30	145	1.72	3	5.33	3.70	16.80	242	5.48	8	0.70	0
Link; Gemini	5.75	0.94	18.01	3.41	0.000	1.64	2.44	12.5	128	1.43	115	1.97	7	5.26	3.36	13.80	243	5.40	6	0.67	1
Stryker; Scorpio	5.77	0.10	18.97	3.53	0.000	1.72	2.33	11.5	116	1.52	130	1.96	4	5.26	3.57	15.80	240	5.47	7	0.40	3
DePuy; Attune	5.94	0.22	18.83	3.54	0.000	0.94	1.54	9.5	123	0.77	121	1.16	6	5.79	3.45	16.80	247	5.86	3	0.20	0
Link; Symphoknee	6.18	0.64	20.18	3.77	0.000	1.18	1.77	9.5	132	1.16	111	1.29	7	5.98	3.68	18.30	247	6.06	1	0.20	2
Ortosintese; Ortosintese	6.44	1.04	16.73	3.49	0.000	2.04	2.46	6.5	128	2.04	120	2.07	2	5.89	3.59	15.80	250	5.89	0	0.00	0
Implantcast; ACS Knee System	6.84	1.62	20.18	3.69	0.000	1.49	2.01	10.5	99	1.20	145	1.74	6	6.54	3.65	17.80	250	6.54	0	0.00	0
Sartori; Sartori	7.10	0.22	18.77	4.02	0.000	2.18	2.89	12.5	125	1.99	123	2.42	2	6.56	3.89	17.40	250	6.56	0	0.00	0
Baumer; Baumer	7.20	0.54	17.68	3.98	0.000	2.05	2.54	8.5	117	2.13	128	2.06	5	6.71	4.03	17.60	250	6.71	0	0.00	0
Vincula; MetaBio	7.58	0.22	18.77	4.23	0.000	2.18	2.90	12.5	128	2.03	120	2.38	2	7.07	4.12	18.40	250	7.07	0	0.00	0
Zimmer; Nex Gen	7.90	0.22	20.70	4.66	0.000	2.93	3.24	10.9	193	3.41	54	1.38	3	7.14	4.27	18.40	244	7.28	5	1.60	1
Impol; NG	8.61	0.94	21.82	4.95	0.000	4.69	5.21	17.5	181	5.27	67	3.27	2	6.90	3.90	18.40	250	6.90	0	0.00	0
Peter Brehm; BPK-S	8.72	2.62	21.15	3.54	0.000	1.24	1.87	10.5	125	1.02	116	1.58	9	8.57	3.41	18.90	250	8.57	0	0.00	0
Orthovasive; Indus	9.22	0.78	22.64	4.97	0.000	4.23	4.34	12.9	184	5.10	63	1.89	3	7.92	4.22	19.40	243	8.14	6	0.45	1
Impol; PAR	9.37	0.99	21.74	4.60	0.000	2.62	3.81	16.5	171	2.76	77	2.37	2	8.75	4.08	20.40	250	8.75	0	0.00	0

Abbreviations: Max, maximum value; Min, minimum value; SD, standard deviation.

**Fig. 2:**
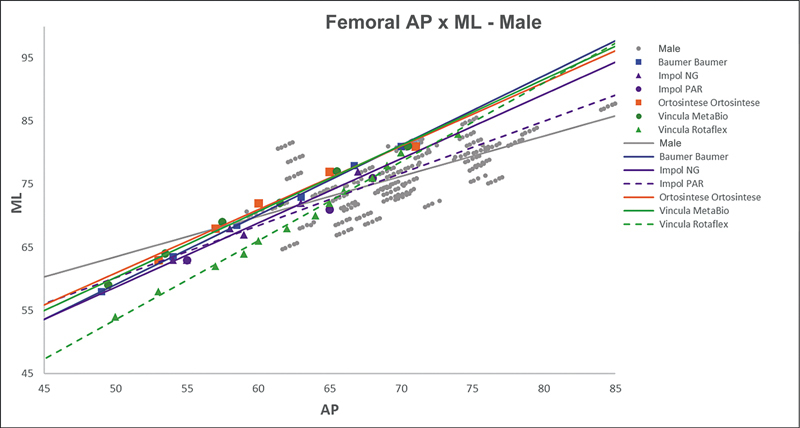
Mediolateral (ML) versus anterolateral (AP) regression lines of femoral measurements in men and Baumer, NG, PAR, Ortosintese, MetaBio, and Rotaflex implants.

Figs. 2^3^,4,5,6,7,8,9 show regression analyses of the world's leading TKA implants, 15 including Brazilian and imported devices, regarding femoral and tibial ML and AP measurements. The results demonstrate that imported implants perform better with morphological “customization,” especially in male knees. The highest discrepancies occurred in the female femurs.

**Fig. 3:**
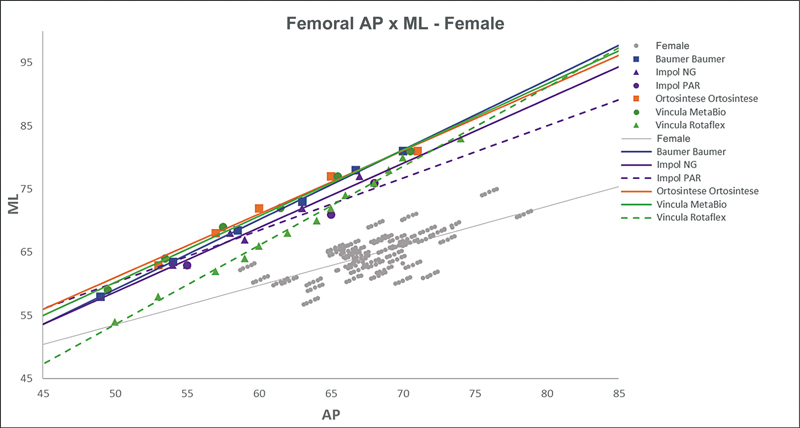
Mediolateral (ML) versus anterolateral (AP) regression lines of femoral measurements in women and the Baumer, NG, PAR, Ortosintese, MetaBio, and Rotaflex implants.

**Fig. 4:**
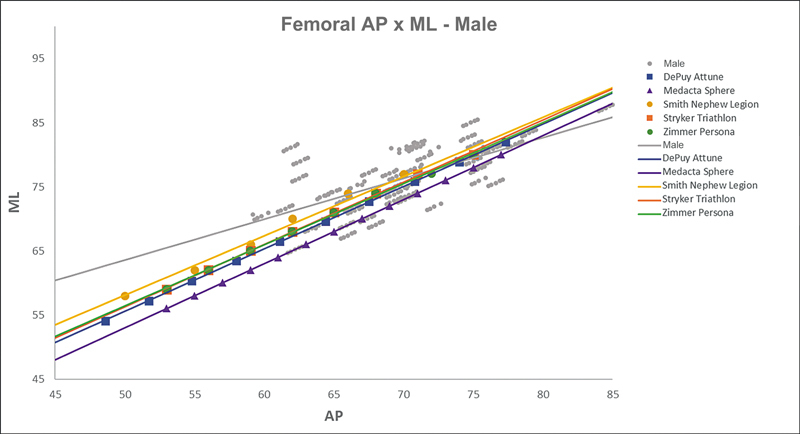
Fig. 4 Mediolateral (ML) versus anterolateral (AP) regression lines of femoral measurements in men and the Attune, Sphere, Legion, Triathlon, and Persona implants.

**Fig. 5:**
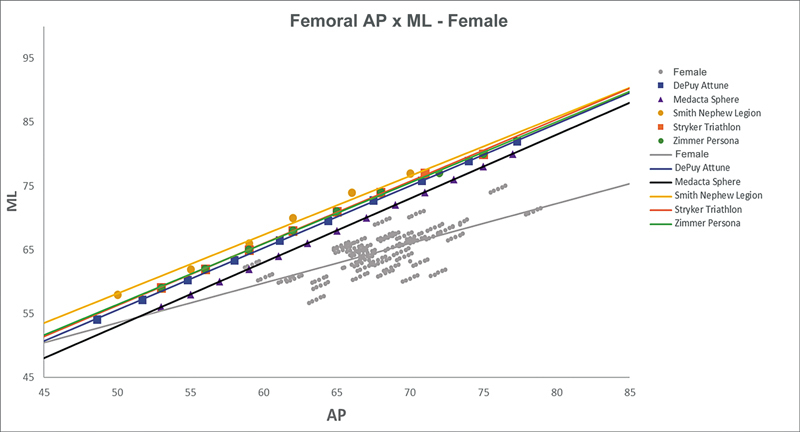
Mediolateral (ML) versus anterolateral (AP) regression lines of femoral measurements in women and the Attune, Sphere, Legion, Triathlon, and Persona implants.

**Fig. 6:**
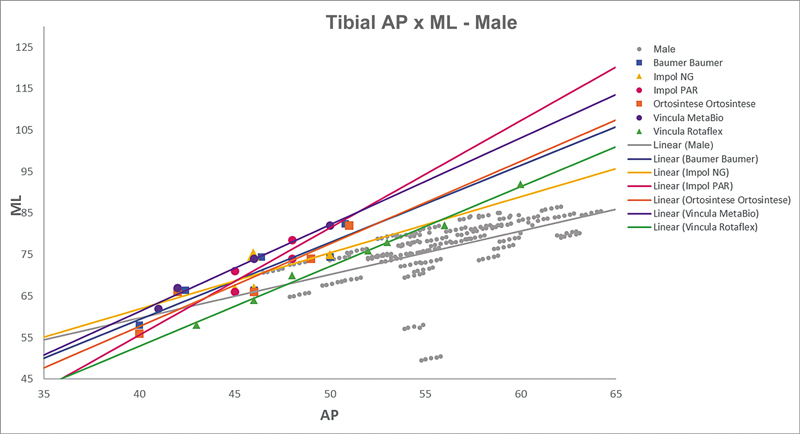
Mediolateral (ML) versus anterolateral (AP) regression lines of tibial measurements in men and the Baumer, NG, PAR, Ortosintese, MetaBio, and Rotaflex implants.

**Fig. 7:**
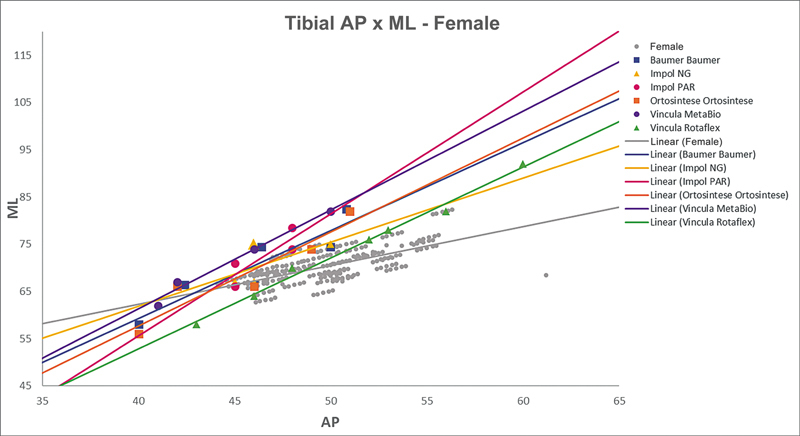
Mediolateral (ML) versus anterolateral (AP) regression lines of tibial measurements in women and the Baumer, NG, PAR, Ortosintese, MetaBio, and Rotaflex implants.

**Fig. 8:**
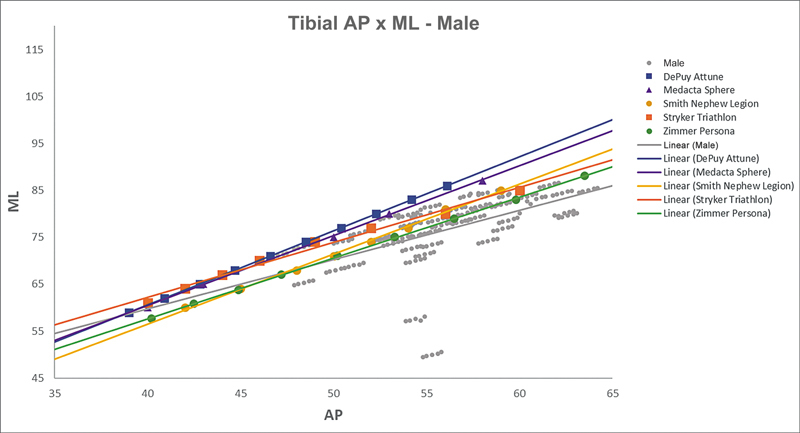
Mediolateral (ML) versus anterolateral (AP) regression lines of tibial measurements in men and the Attune, Sphere, Legion, Triathlon, and Persona implants.

**Fig. 9:**
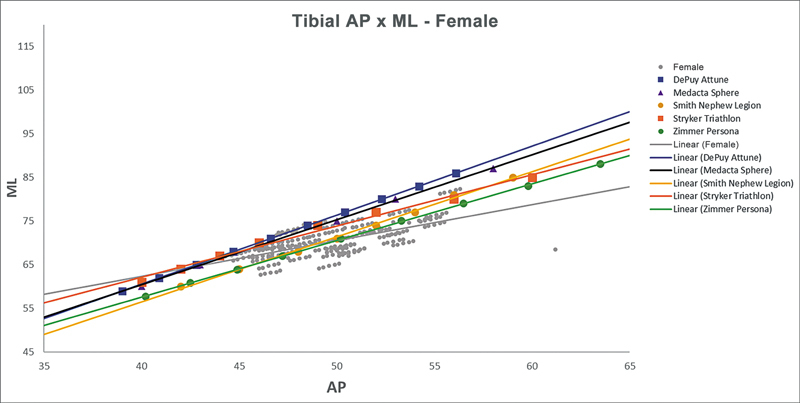
Mediolateral (ML) versus anterolateral (AP) regression lines of tibial measurements in women and the Attune, Sphere, Legion, Triathlon, and Persona implants.

For women, considering AP/ML accommodation for TKA devices, the U2 implant had the best statistical score, followed by the Columbus and Legion implants ( Table 6 ). For men, the best devices were Persona, Advance, and Legion ( Table 7 ).

**Table 6:** Implant ranking for placement in female patients

Manufacturer; prosthesis	Final score	Final score calculation					
		Tibial values			Femoral values		
		Euclidian distance	Ranking	Normalized value	Euclidian distance	Ranking	Normalized value
United Orthopedic; U2	0.11	2.66	3	0.11	2.36	2	0.00
Aesculap; Columbus	0.14	2.74	4	0.13	2.39	4	0.00
Smith & Nephew; Legion	0.27	2.19	1	0.00	3.97	16	0.27
Maxx Orthopedics; Freedom	0.20	2.76	5	0.14	2.76	7	0.07
Zimmer; Persona	0.27	2.54	2	0.08	3.47	13	0.19
Meril; Freedom	0.25	2.92	7	0.17	2.79	8	0.07
Vincula; Rotaflex	0.48	2.83	6	0.15	4.31	19	0.33
Microport; Advance	0.45	3.37	9	0.28	3.36	11	0.17
Medacta; Sphere	0.39	3.81	19	0.39	2.36	1	0.00
Link; Gemini	0.48	3.95	21	0.42	2.72	6	0.06
Stryker; Triathlon	0.53	3.63	10	0.35	3.49	14	0.19
DePuy; Attune	0.51	3.81	18	0.39	3.09	9	0.12
Orthovasive; Indus	0.55	3.63	11	0.35	3.61	15	0.21
DePuy; Sigma	0.58	3.85	20	0.40	3.45	12	0.18
Link; Symphoknee	0.52	4.35	23	0.52	2.39	3	0.00
Stryker; Scorpio	0.65	3.71	16	0.37	4.06	17	0.28
Implantcast; ACS Knee System	0.66	4.83	25	0.63	2.53	5	0.03
Zimmer; Nex Gen	0.99	3.31	8	0.27	6.66	22	0.72
Impol; NG	0.96	3.68	13	0.36	5.99	21	0.61
Impol; PAR	0.96	4.41	24	0.53	4.88	20	0.42
Sartori; Sartori	1.16	3.68	14	0.36	7.14	23	0.80
Baumer; Baumer	1.22	3.66	12	0.35	7.56	24	0.87
Vincula; MetaBio	1.26	3.70	15	0.36	7.73	25	0.90
Ortosintese; Ortosintese	1.39	3.80	17	0.39	8.35	26	1.00
Peter Brehm; BPK-S	1.31	6.35	26	1.00	4.22	18	0.31

**Table 7:** Implant ranking for placement in male patients

Manufacturer; prosthesis	Final score	Final score calculation					
		Tibial values			Femoral values		
		Euclidian distance	Ranking	Normalized value	Euclidian distance	Ranking	Normalized value
Zimmer; Persona	0.01	3.27	1	0.00	3.55	2	0.01
Microport; Advance	0.05	3.38	2	0.02	3.63	3	0.03
Smith & Nephew; Legion	0.08	3.50	3	0.04	3.68	6	0.04
Aesculap; Columbus	0.23	4.34	4	0.18	3.73	7	0.06
Stryker; Triathlon	0.18	4.39	6	0.18	3.52	1	0.00
Vincula; Rotaflex	0.37	4.36	5	0.18	4.24	14	0.19
United Orthopedic; U2	0.39	4.72	7	0.24	4.08	9	0.15
Medacta; Sphere	0.51	5.28	8	0.33	4.19	12	0.18
Meril; Freedom	0.50	5.49	9	0.36	4.03	8	0.14
Maxx Orthopedics; Freedom	0.55	5.49	10	0.36	4.21	13	0.18
DePuy; Attune	0.47	5.94	14	0.44	3.65	5	0.04
Link; Symphoknee	0.51	6.18	15	0.48	3.64	4	0.03
Link; Gemini	0.70	5.75	12	0.41	4.64	15	0.30
Stryker; Scorpio	0.76	5.77	13	0.41	4.85	16	0.35
Implantcast; ACS Knee System	0.74	6.84	18	0.59	4.10	10	0.15
DePuy; Sigma	1.12	5.74	11	0.41	6.21	23	0.71
Ortosintese; Ortosintese	1.04	6.44	17	0.52	5.49	18	0.52
Sartori; Sartori	1.08	7.10	19	0.63	5.24	17	0.46
Peter Brehm; BPK-S	1.06	8.72	24	0.89	4.15	11	0.17
Baumer; Baumer	1.22	7.20	20	0.64	5.70	21	0.58
Vincula; MetaBio	1.24	7.58	21	0.71	5.54	19	0.53
Impol; NG	1.51	8.61	23	0.88	5.92	22	0.63
Impol; PAR	1.56	9.37	26	1.00	5.64	20	0.56
Zimmer; Nex Gen	1.63	7.90	22	0.76	6.80	24	0.87
Orthovasive; Indus	1.98	9.22	25	0.98	7.30	25	1.00

## Discussion

Manufacturers of TKA commonly use specific population groups as references for developing their implants. Although their sample may have some ethnic diversity, selected populations can present with distinct knee characteristics, contributing to divergences in comparison with other ethnic groups. 10 13 16

The results demonstrated that, on average, the morphological variations between implants and knees from Brazilian patients do not pose a major concern to surgeons regarding two-dimensional femoral and tibial sizing. However, we noted differences greater than 10 mm in patients from both genders, in several implants. In such cases, the surgeon should consider modifying implant sizing for better anatomical accommodation. These discrepancies directly influence the knee's biomechanical conditions, as they can interfere with the extension and flexion joint spaces as well as the ability to interchange different femoral and tibial implant sizes, especially for devices that require resection of the PCL.

The biomechanical importance of the femoral implant AP measurement stood out due to its influence on knee stability and direct correspondence with the dimensioning of the flexion space. Increasing or decreasing the posterior offset of the femoral component can directly alter the flexion space and influence the extension space. This occurs because the relationship between the capsular contact and the posterior soft tissues can contribute to the decrease or increase of extension space. 17 18 Our results demonstrated that, in women, no femoral implant presented maximum variations for mean divergences greater than 3 mm for the AP measurement. However, four implants presented mean AP divergences greater than 3 mm in men (PAR, NG, Nex Gen, Indus). Therefore, femoral components require greater planning due to the influence on joint spaces if the AP dimension requires modification, especially in Brazilian men.

Surgeons must be aware of the risk of notching in the anterior cortex when the AP divergence is greater than 3 mm, given the higher risk of periprosthetic fractures. 19 No female femoral implant presented an average divergence greater than 3 mm. However, the results in male femurs require better attention, since 10 implants (Scorpio, Sartori, Ortosintese, MetaBio, PAR, Baumer, NG, Sigma, Nex Gen, Indus) had divergences to bone samples greater than 3 mm compared with the best AP implant, increasing the risk of invasion of the cortical section depending on the type of guide used in the procedure.

Regarding the discrepancy in femoral implant accommodation for the ML measurement, the greater clinical relevance occurred in women, with mean values for ideal coverage varying by 4.22 mm. This indicates that the studied implants have a less “customized” ML accommodation ratio for the femurs of Brazilian women than men. The results reinforce the need for narrow implants to benefit this population. The presence of implants with mediolateral augmentation is a significant clinical fact, as surgeons might need to undersize them to avoid contact with periarticular capsuloligamentous structures, leading to potential postoperative pain. It is noteworthy that undersizing a femoral implant for mediolateral accommodation directly influences joint space size, especially for flexion.

The ML ratio of male femurs was wider compared with female ones. McNamara et al. reported this same characteristic in a Hispanic population. 12 Given these findings, it would be appropriate to have narrow implants that support the same AP distance with a smaller ML dimension, as well as wide implants with the same AP distance and more elongated ML dimension. These would provide better bone accommodation for each patient, as the lack of mediolateral femoral bone coverage leads to support distant from the cortices, such as in cancellous bone with lower mechanical resistance. Support in cancellous bone could cause complications, such as femoral and tibial subsidence.

It is worth noting that the mean differences for the ML distance were usually below 5 mm. This distance in the medial and lateral compartments does not have clinical relevance to the outcome, being accepted as implant under- or oversizing with no technical compromise to the femur and tibia.

In our study, tibial implants had a better fit in female than male anatomy, mainly due to the AP distance. Although it may decrease posterior cortical support and, as a result, expose the implant to cancellous bone, there was no concern for oversizing, in which the implant contacts the posterior capsular and tendinous structures, potentially causing postoperative discomfort and pain. 20 21

Several studies on knee morphological aspects reported satisfactory anatomical adaptation outcomes. Yue et al. 22 and Cheng et al. 5 9 reported the proximal tibial anthropometric relationships of Asian subjects and compared them with findings from other populations, demonstrating joint morphological differences. Also, Vaidya et al., 23 Urabe et al., 24 and Chaichankul et al. 25 analyzed the correlations between TKA components and epiphyseal morphologies in Indian, Japanese, and Thai populations, and reported good measurement correspondence. Thus, although anatomical variations in different global populations compromise the full customization of TKA implants, the available sizes often meet bone coverage and functionality requirements.

Nevertheless, Ho et al. 26 found that the relation of some implants may not be satisfactory for the Chinese population. In the AP versus ML dimensional ranking for both the femoral and tibial components, imported implants stood out as they showed the best proportions for both genders. Regarding Brazilian devices, the Rotaflex implant stood out, ranking seventh and sixth for both women and men. In this context, there is an opportunity to optimize the morphological development of Brazilian implants to improve joint fit for our population.

The present study has some limitations, and its results require a critical analysis. We did not assess the patients' height and weight (biometric profile), which hindered our understanding of whether the anatomical differences in the population might result from other biotypical characteristics, as well as joint size variations that could influence customization according to each manufacturer's component numbers.

Furthermore, we did not analyze the ethnicity profile of our sample to determine whether there is some race or color-related bias. The bone sections standardized in the radiological study may not correspond to operative ones, depending on surgical conditions (for instance, hypoplasia or bone defects). This divergence might have added a transposition bias to our results.

Another limiting factor of the study was the lack of evaluation of lateral AP tibial measurements in asymmetrical implants. Although anatomical implants may offer better lateral accommodation than nonanatomical implants, we did not analyze this fact.

Moreover, we did not analyze the interchangeable correlation between femoral and tibial implants for cases of bone sizing discrepancies.

Our ranking corresponds to the morphological findings of the analyzed sample and may not represent the entire Brazilian population.

## Conclusion

The implants studied provided satisfactory bone coverage in the knees of the Brazilian population, mostly with small variations in dimension. However, we observed some differences greater than 10 mm. Imported implants proved to be more customizable than Brazilian implants in the Brazilian population.
